# Solitary perturbations in the steep boundary of magnetized toroidal plasma

**DOI:** 10.1038/srep45075

**Published:** 2017-03-24

**Authors:** J. E. Lee, G. S. Yun, W. Lee, M. H. Kim, M. Choi, J. Lee, M. Kim, H. K. Park, J. G. Bak, W. H. Ko, Y. S. Park

**Affiliations:** 1 Pohang University of Science and Technology, Pohang 790-784, Korea; 2 National Fusion Research Institute, Daejeon 34133, Korea; 3 Ulsan National Institute of Science and Technology, Ulsan 689-798, Korea; 4 Columbia University, New York, New York 10027, USA

## Abstract

Solitary perturbations (SPs) localized both poloidally and radially are detected within ~100 *μ*s before the partial collapse of the high pressure gradient boundary region (called pedestal) of magnetized toroidal plasma in the KSTAR tokamak device. The SP develops with a low toroidal mode number (typically unity) in the pedestal ingrained with quasi-stable edge-localized mode (QSM) which commonly appears during the inter-collapse period. The SPs have smaller mode pitch and different (often opposite) rotation velocity compared to the QSMs. Similar solitary perturbations are also frequently observed before the onset of complete pedestal collapse, suggesting a strong connection between the SP generation and the pedestal collapse.

High-confinement mode (H-mode) regime of toroidal magnetized plasma, which is the main operational regime for modern tokamak reactors, is characterized by improved energy and particle confinement connected to the formation of an edge transport barrier (also called edge pedestal)^1^. The resulting steep pressure gradient of the pedestal provides a free energy source for a class of radially-localized magnetohydrodynamic (MHD) instabilities called edge localized mode (ELM)^2^. The ELM structure has been observed in the low field side of the tokamak plasma like poloidal array of filaments, i.e., field aligned tubes with finite poloidal wavelength. The pedestal undergoes a rapid collapse as the ELM filament bursts and the same process repeats quasi-periodically. Note that in this paper, ELM indicates the filamentary mode structure in the pedestal and not the collapse event of the pedestal. The ELM burst or crash is used as metonym to pedestal collapse owing to their temporal proximity and rapidness.

The repetitive flux of energy and particles released by the ELM crash can cause severe damage at the strike points on the plasma facing components (PFCs)^3^. The control of the ELM crash or pedestal collapse is therefore a critical issue for successful operation of international thermonuclear experimental reactor (ITER) and future fusion reactors^4^. However, the trigger mechanism of the crash onset has not been identified yet. ELM is regarded as a precursor to the crash in many tokamaks^5^,^6^ but it is unclear whether they immediately trigger the crash. On the Korea Superconducting Tokamak Advanced Research (KSTAR), quasi-stable edge-localized filamentary modes (QSMs) and their complex structural transitions without crash are routinely observed in H-mode discharges^7^,^8^ using 2D electron cyclotron emission (ECE) imaging diagnostics^9^. These observations suggest QSMs are not directly linked to the crash trigger.

Recently, solitary magnetic perturbation (SMP) has been observed within ~±100 *μ*s from the onset of the pedestal erosion, suggesting it as a possible crash trigger^10^,^11^. The SMPs are strong magnetic perturbations well localized in poloidal and radial direction close to the separatrix (the last closed flux surface of the toroidal plasma). The perturbations have low toroidal mode numbers and generally propagate in the electron diamagnetic direction. The poloidal and radial components of SMPs have temporally even and odd symmetries, respectively. Based on magnetic field measurements, two models were proposed for the SMPs: bi-polar current filament moving along the poloidal direction^10^ and mono-polar current filament with ratio of poloidal to radial propagating velocity ~0.75^11^.

On the KSTAR, solitary edge-localized perturbations (SPs) similar to SMPs are frequently observed within ~100 *μ*s before the onset of partial pedestal collapse. The SPs are strongly localized in poloidal and radial direction with low toroidal mode numbers (typically unity). The perturbation structures are clearly captured by both magnetic probes such as Mirnov coils^12^ and the ECE imaging diagnostics measuring the fluctuations of the local ECE radiation temperature (*T*_ece_). The SPs appear approximately at the same flux surface (inside the separatrix) where QSMs are observed during the inter-crash period. The SPs propagate in electron diamagnetic direction in most cases and are easily distinguished from QSMs by spatial structure, amplitude and flow velocity. Solitary perturbation structures have been also observed at the onset of complete pedestal collapse (similar to the irregular filamentary structure reported in ref. 7). However, it is difficult to resolve the SP structure in the case of the complete collapse because of large variations in the diagnostic signals during the complete collapse overwhelming the signals cresponding to the SP. Therefore, this paper focuses on the SPs associated with partial pedestal collapse in order to describe the structure and dynamics with clarity. Before detailed description of SPs, the phenomenology of partial ELM crash is explained based on the deuterium alpha (*D*_α_) line radiation, radio-frequency (RF) electromagnetic emission bursts and changes of radiation temperature outside the separatrix. Description of the SPs is followed based on measurements using 3D ECE imaging (ECEI) diagnostic^13^ and toroidal Mirnov coil array.

## Results

### Partial ELM crash

The ELM crash is typically accompanied with *D*_α_ radiation spike and rapid reduction of plasma parameters such as radiation temperature and density in the edge and plasma stored energy^2^. Another accurate indicator of the ELM crash is strong electromagnetic emission in the radio frequency (RF) range^14^, which may be interpreted as ion cyclotron emission (ICE) attributed to the magnetoacoustic cyclotron instability (MCI) excited by the increase in the population of fusion-born particle at the edge of plasmas^15^. When the ELM crash occurs, the edge pedestal collapses with the ejection of large energy and particles from the plasma. After the crash, the edge pedestal profiles recover and build up to the next crash. The partial ELM crash, which is the focus of this paper, is incomplete collapse of the edge pedestal during the recovery phase. At the partial crash, RF bursts, weak *D*_α_ radiations are observed while there is no noticeable decrease in the stored energy.

**Figure 1:**
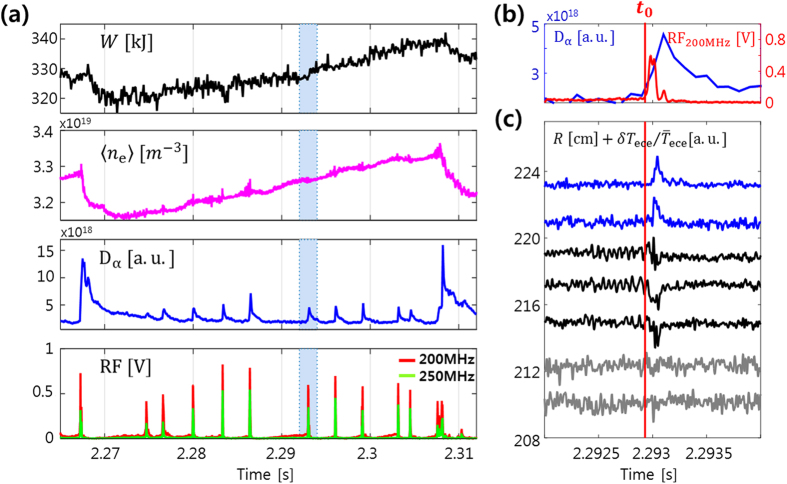
Characteristics of the partial ELM crash. (**a**) Time traces of stored energy (black), line-averaged density (magenta), *D*_α_ emission (blue), and RF signals for 200 MHz (red), 250 MHz (green) are shown for shot no. 13250. (**b**) Time traces of the *D*_α_ emission (blue) and 200 MHz RF emission (red) for the partial ELM crash are highlighted in (**a**). *t*_0_ is the start time of the RF burst. (**c**) Time traces of 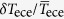 at the edge region are compared. Gray, black and blue colors indicate the signal locations inside the pedestal, on the pedestal and outside the separatrix, respectively.

An example of partial ELM crash in shot no.13250 is illustrated in Fig. 1. A brief description of the discharge is as follows: the toroidal magnetic field *B*_T_ = 1.8 T, the plasma current *I*_p_ ~ 600 kA, the stored energy *W* ~ 330 kJ, the line-averaged electron density <*n*_e_> ~ 3.3 × 10^19^ *m*^−3^, the elongation factor *κ* ~ 1.68, and the edge safety factor calculated by equilibrium fitting (EFIT) code^16^ *q*_95_ ~ 4.3. This plasma is lower single null configuration and heated by ~2.7 MW neutral beam injection (NBI). Figure 1(a) shows the time traces of the plasma stored energy, <*n*_e_>, *D*_α_ emission from the divertor and RF signals at 200 MHz and 250 MHz. A complete ELM crash occurs at ~2.268 s with decrease of the plasma stored energy (~10 kJ) and the line-averaged electron density (~10^18^ *m*^−3^) and strong bursts of the *D*_α_ emission and RF signals. Before the next ELM crash ~41 ms later, several small peaks are observed in the *D*_α_ emissions. These peaks are synchronized with strong RF peaks. On the other hand, there are no noticeable drops in the stored energy and the density. Such transport events without substantial change of the stored energy and the density are termed as the partial ELM crash. Figure 1(b) shows the *D*_α_ emission and 200 MHz RF signal around the partial ELM crash marked by the blue box in Fig. 1(a). The start time of the rise of the RF signal is indicated as *t*_0_ (2.292945 s). The *D*_α_ emission starts to increase almost at the same time. Figure 1(c) shows the time traces of the *T*_ece_ fluctuations at different radial locations in the edge region measured by one of two ECEI diagnostics^9^,^13^. Gray, black and blue traces correspond to the locations inside the pedestal, on the pedestal, and outside the separatrix, respectively. The increase of the *T*_ece_ fluctuations outside the separatrix (blue traces in Fig. 1(c)) is due to the ejection of heat from the plasma.

**Figure 2:**
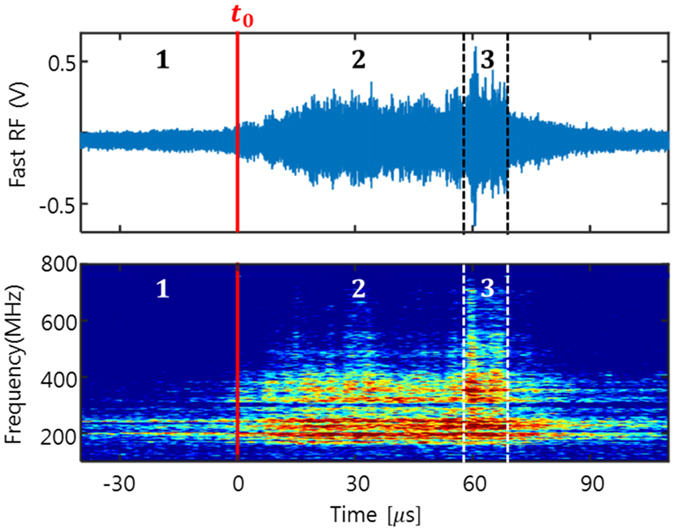
Three distinct regions in the RF spectrogram for partial ELM crash. (1) narrow-band RF spectral lines around 190 MHz and 240 MHz, (2) spectrum broadened up to ~500 MHz with many discrete spectral lines, and (3) intense RF burst signal. The edge pedestal starts to collapse at *t*_0_ + 60 *μ*s.

The fast RF spectrometer with 5 GS/s sampling rate^14^ is also used to capture the spectral features of the RF burst at the partial ELM crash. Figure 2 shows the time trace and spectrogram for the RF signal around *t*_0_. There are three distinct regions in the RF spectrogram: In the first region (*t* < *t*_0_), the RF signal has narrow-band spectral lines around 190 MHz and 240 MHz approximately corresponding to the lower hybrid resonance frequency at the plasma edge. In the second region (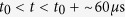), the spectrum becomes stronger and broadened up to ~500 MHz with discrete harmonic ion cyclotron emission (ICE) lines. In the third region (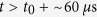), an intense RF burst signal appears with broader spectrum from 160 MHz to 800 MHz. The intense RF burst coincides with the pedestal collapse measured by the ECEI. These distinct spectral features of the partial ELM crash are similar to the case of complete ELM crash^14^, suggesting a common crash mechanism.

### Observation of solitary perturbation

Within ~100 *μ*s before partial collapse, solitary perturbation structures are often detected by the ECEI system. The SPs are distinguished from the QSMs in spatial structure, amplitude and flow velocity. The toroidal mode numbers of the SPs are typically unity and the radiation temperature fluctuations associated with the SPs are characterized by isolated peaks and dips with large amplitude.

**Figure 3:**
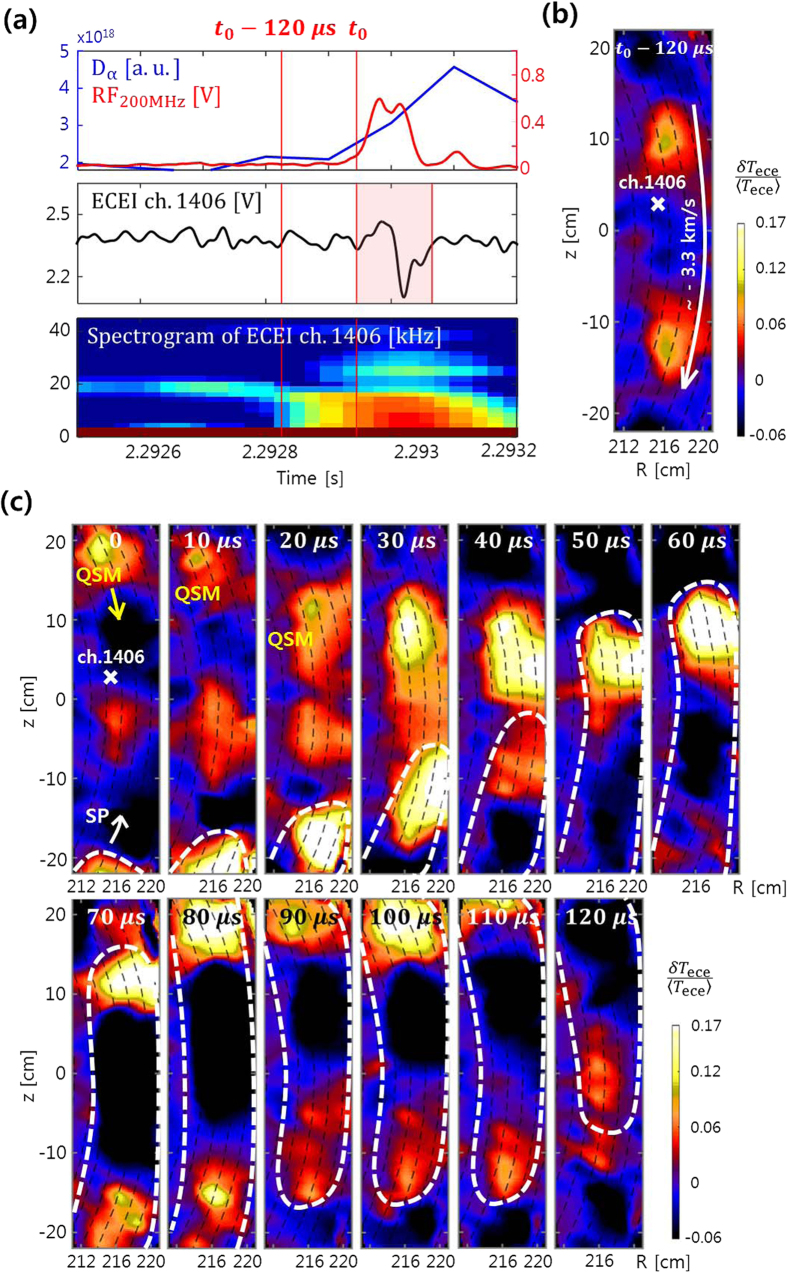
Comparison of dynamics between SP and QSM (see supplementary video). (**a**) Time traces of *D*_α_ emission (blue), 200 MHz RF signal (red), ECEI signal (ch.1406) and its spectrogram. The middle ECEI time trace is band-pass filtered from 3 to 45 kHz for clarity. (**b**) ECEI image of the QSM structure rotating ~−3.3 km/s at *t*_0_ − 120 *μ*s. (**c**) Consecutive frames of ECEI images in the time period highlighted in (**a**). While the QSM moves with ~−2.8 km/s, the SP indicated by white dashed lines moves with ~+5.9 km/s.

Figure 3 represents dynamics of the SP and QSM measured by the 2^nd^ ECEI system for the partial ELM crash highlighted in Fig. 1(a). The time trace and spectrogram of *T*_ece_ fluctuation at the mode location are shown in Fig. 3(a) along with *D*_α_ emission and 200 MHz RF signal. The QSM structure with the mode frequency *f*_mode_ ~ 20 kHz appears inside the separatrix ~600 *μ*s before *t*_0_. The mode frequency decreases toward the partial ELM crash. Figure 3(b) is an image of the QSM at *t*_0_ − 120 *μ*s. The poloidal wavelength of the mode is 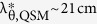 and the apparent poloidal velocity is 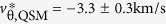 (the negative sign indicates the clockwise direction or the ion diamagnetic direction). The filamentary mode moves along the magnetic flux surfaces (black dashed lines calculated by EFIT code). The toroidal mode number (*n*) estimated from the ECEI image^17^ is 11 ± 1. Note that 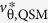 was ~−4.3 km/s at the beginning and slowed down to ~−2.8 km/s toward the partial ELM crash while maintaining the poloidal spacing between the filaments.

From *t*_0_, the ECEI signal shows a large dip (highlighted in Fig. 3(a)) with the corresponding broad spectrum up to ~42 kHz. A distinct solitary perturbation structure is clearly captured as indicated by white dashed lines in Fig. 3(c). This SP has a well localized structure in the poloidal direction unlike the QSM. The motion of SP is discontinuous with intermittent pauses. The apparent poloidal velocity of SP 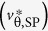 measured by the ECEI system is on average +5.9 ± 0.3 km/s (the positive sign indicates the counter-clockwise direction or the electron diamagnetic direction). The SP propagates in the electron diamagnetic direction in most cases but also in the ion diamagnetic direction in rare cases. The first frame (*t*_0_) shows the QSM structure moving downward (clockwise) in the upper region and the SP structure moving upward (counter-clockwise) in the bottom region. The radial positon of SP is ~218 cm in the outboard mid plane, which is slightly more external than the radial position of the QSM 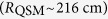. They overlap around 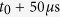 and move together in the counter-clockwise direction. Note that the SP has a relatively faster speed and higher amplitude compared to the QSM. The flow directions of SP and QSM are not always opposite but the speeds of these two modes generally differ noticeably.

**Figure 4:**
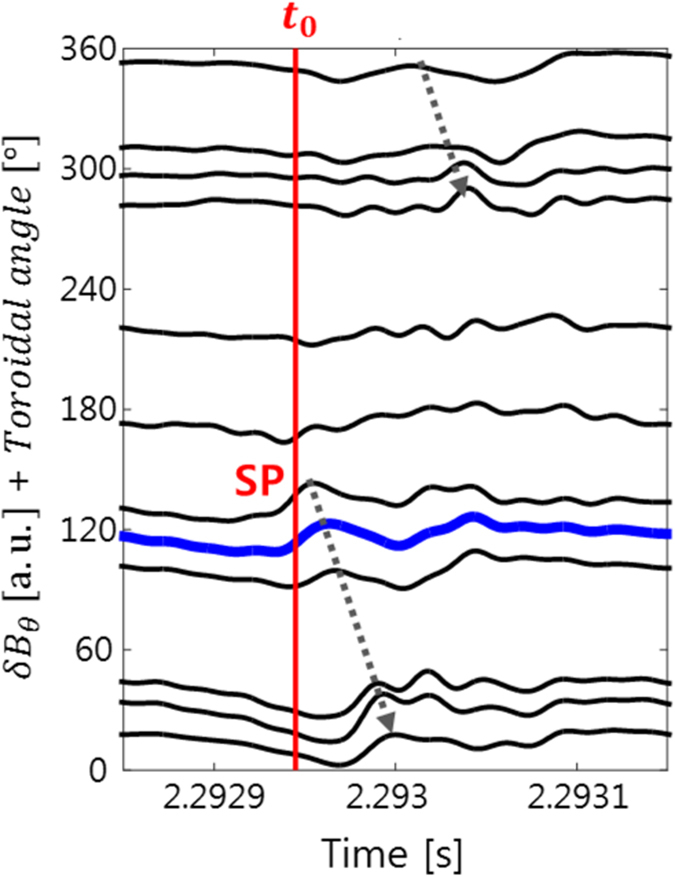
Poloidal magnetic field fluctuations corresponding to SP. Poloidal magnetic fields are obtained by integrating the Mirnov coil signals. The bold blue line corresponds to approximately the same toroidal position as the 2^nd^ ECEI system. Gray arrows indicate the direction of apparent toroidal velocity of the SP.

It is expected that the SP may carry currents involving magnetic field perturbations. Figure 4 shows the time traces of the poloidal magnetic field fluctuations (δ*B*_θ_) measured by the toroidal array of Mirnov coils. The poloidal installation angle of the Mirnov coils is ~56° from the outboard mid plane where the ECEI systems are installed. In order to directly compare the Mirnov data to the ECEI data, the Mirnov signals are mapped to the mid plane along the magnetic field lines. The toroidal angle (*ϕ*) increases starting from the L port (*ϕ* = 0°) in the counter-clockwise direction from the top view of the KSTAR tokamak. The bold blue line corresponds to the Mirnov coil located at approximately the same position as the 2^nd^ ECEI system (*ϕ* = 112.5°). Around *t*_0_, a strong magnetic field is observed near *ϕ* ~ 130°. The apparent toroidal velocity of SP (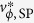) measured by the Mirnov coils is +92 ± 2 km/s (indicated by gray arrows). The number of peaks (valleys) per toroidal turn at a given time is 1, i.e., *n* = 1. Two distinct SPs with *n* = 1 moving in the opposite directions from each other or SPs with *n* = 2 have been observed in some cases.

### Pitch angle of solitary perturbation

**Figure 5:**
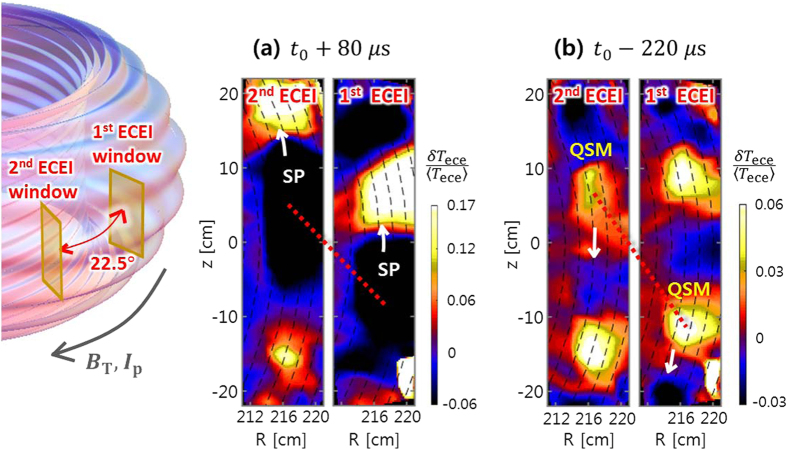
3D structures of SP and QSM. (**a**) SP structure rotating in the counter-clockwise direction with pitch angle ~7.6°. (**b**) QSM structure rotating in the clockwise direction with pitch angle ~10.5°. The red dotted lines indicate the mode structure along the same flux tube in the two ECEI views.

The SP was simultaneously observed by the 1^st^ ECEI system (toroidally separated by 22.5 degrees from the 2^nd^ ECEI system), which provides information on the 3D structure of the SP. The quasi 3D images strongly support the existence of SP before the partial ELM crash. The mode pitch at the outboard mid plane (α^*^) can be calculated by measuring the vertical distance of the same flux tube in the 3D images (Δ*z*) given the fixed toroidal distance between two ECEI systems^17^. Figure 5(a) shows the upward movement of the SP structures detected by two ECEI systems at 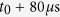. For comparison, Fig. 5(b) shows the QSM structure moving downward at 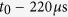. The red dotted lines indicate the same flux tubes (perturbation structures) observed in each ECEI system. The pitch angle of the SP at the outboard mid plane (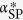) is 6.6 ± 1° (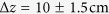), and is significantly reduced compared to the pitch angle of the QSM at the outboard mid plane (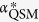) which is 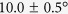 (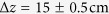). It is difficult to explain the large decrease in mode pitch for a short time period (~few tens of *μ*s) by any conceivable change of current profile such as bootstrap current formed in the pedestal^18^. The substantial reduction of the pitch may suggest that the SP structure is not aligned with the magnetic field lines, which will be addressed in more detail at the discussion section.

### Velocity of solitary perturbation

The mode velocity can be decomposed into the parallel and perpendicular velocity with respect to the mode pitch. If the mode structures are highly elongated, the parallel velocity of the mode (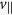) has no contribution to the apparent velocities in the lab frame. Therefore, the apparent toroidal and poloidal velocities are determined by the perpendicular velocity (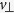) and pitch angle of the mode:


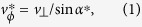



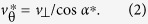


The perpendicular mode velocity can be expressed as


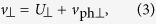


where 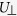 and 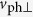 are the plasma bulk flow and phase velocity in the perpendicular direction, respectively. Using the toroidal and poloidal basis, 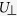 can be written as


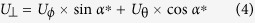


where *U*_*ϕ*_ and *U*_θ_ are the bulk toroidal and poloidal plasma velocities, respectively.

The apparent velocities of the SP are difficult to understand at a glance: (1) 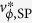 is opposite to *U*_*ϕ*_ and (2) 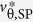 differs greatly from 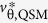 while the difference in the radial position is small. These peculiarities provide useful information on the plasma flow and the radial electric field at the pedestal.

**Figure 6:**
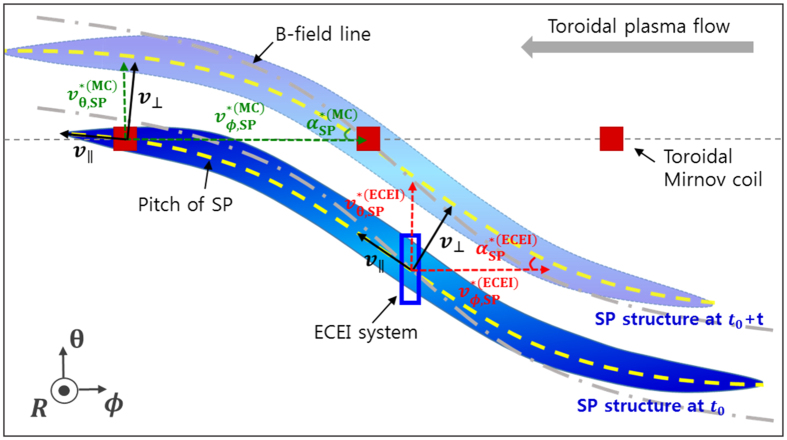
Schematic illustration of SP structure. The ECEI system and the toroidal Mirnov coils are indicated by the blue empty box and red filled boxes, respectively. The gray arrow on the top right indicates the direction of the toroidal plasma flow. The thick blue curve and the pale blue curve represent the SP structure and its motion. The yellow dashed lines indicate the pitch of the SP, which is different from the pitch of the magnetic field lines (gray dash-dot lines).

Figure 6 shows a schematic illustration of SP structure. As the SP structure moves in the laboratory frame, 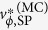 and 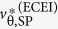 can be measured by the toroidal Mirnov coil array and the ECEI system, respectively. The superscripts MC and ECEI are explicitly used to indicate the measurement locations: e.g. 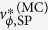 is the apparent toroidal velocity of SP at the poloidal angle of the Mirnov coils. It is interesting to note that the large difference between 
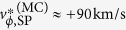
 and the bulk toroidal flow 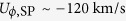 measured by charge exchange spectroscopy (CES)^19^. Using the equations (1), (3) and (4), 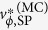 can be written as


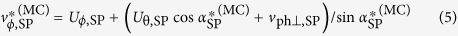


The apparent toroidal velocity of SP can appear opposite to the bulk plasma flow if the second term on the right hand side is large enough. The second term can be approximated using the measurements 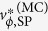 and 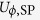
with approximations 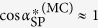 and 
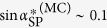
 (the latter conditions comes from the observation 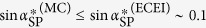):


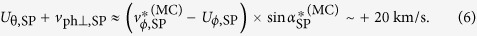


The difference between the apparent poloidal velocities of SP and QSM,


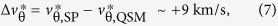


is substantial considering the small difference in the radial positions Δ*R* ~ 2 cm, which indicates a strong flow shear. Using the small pitch angle approximation,


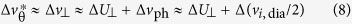


where the phase velocities are assumed close to that of the ballooning mode, i.e., half the ion diamagnetic velocity 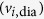
20. The radial electric field is given by ref. 21


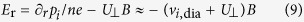


Combining equations (8) and (9) with 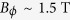 at the mode position yields


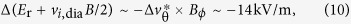


This indicates that the large flow difference (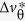) is caused by either *E*_r_ gradient and/or diamagnetic velocity gradient.

## Discussion

This paper focuses on the solitary perturbations observed right before the partial ELM crash or partial collapse of the edge pedestal rather than the complete ELM crash which is more violent and difficult to describe clearly. The partial ELM crash is identified by the D-alpha radiation, RF burst, increasing radiation temperature outside the separatrix, and the distinct spectral change of the fast RF signal. Within ~100 *μ*s before the partial ELM crash, the SP structures have been clearly detected by the 3D KSTAR ECEI system and the toroidal Mirnov coil array. The perturbation is poloidally and radially localized with large amplitude. It propagates mostly in the electron diamagnetic direction and has a low toroidal mode number (typically unity). The apparent poloidal velocity of SP is generally faster than the velocity of the QSM and the mode pitch of SP is noticeably smaller than that of QSM.

The mode pitch of the SP may be interpreted in two ways: (1) The SP is aligned with the magnetic field lines and the magnetic pitch decreases at larger radii in the low field side due to a localized bootstrap current. (2) The SP is not aligned with the magnetic field lines and has a smaller mode pitch compared to the magnetic field pitch. In the first case (field-aligned SP), it is difficult to explain the large decrease in mode pitch (~3.5°) from QSM to SP for a short time period (~few tens of *μ*s). The larger radial position of SP compared to the radial position of QSM may only account less than 1° reduction. Note that a very large increase of the bootstrap current (~200 kA) in the pedestal region might account for ~3.5° pitch reduction, which seems not plausible. Therefore, it is reasonable to assume that SP structure is not aligned with the magnetic field lines, i.e. breakdown of the frozen-in condition. The mode pitch can be changed by a radial displacement induced by poloidally localized quasi-electrostatic perturbation^22^. Assuming the SP is a current-carrying flux tube and the radial displacement occurs quasi-statically, the pitch of the SP will be smaller than the magnetic field pitch to satisfy the single fluid force balance, 
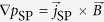
.

An irregular mode structures have been frequently observed near the ELM crash by the KSTAR ECEI system^7^. This structure emerges as one dominant filament with large amplitude after the QSM disappears. In addition, the tongue-shaped deformation of magnetic field, which was theoretically predicted in the low field side of toroidal plasma^23^, was observed before an onset of large-scale collapse event in magnetized plasmas^24^. The tongue structure is localized in three (toroidal, poloidal and radial) directions. These observations, which are similar to the SP near the collapse event, suggest that SP is strongly connected to the crash onset.

The observed SPs may be compared to nonlinear growth of a low-*n* mode reported in numerical simulations of the ELM crash phase^25^,^26^,^27^. In those numerical studies, the energy of the low-*n* mode (typically *n* = 1) rapidly grows and becomes comparable to the energies of the linearly dominant high-*n* modes in the nonlinear phase due to the nonlinear mode coupling. It remains a future work to identify the SP as a low-*n* mode generated by the nonlinear interaction between high-*n* modes. Our observations provide solid experimental data for validation of theory and numerical simulations and pave a way to identification of the governing equations for the SP generation and pedestal collapse mechanisms. The SPs in the plasma boundary layer may also be a general interest as a strong nonlinear boundary phenomenon.

## Methods

### Korea superconducting tokamak advanced research

The KSTAR is a tokamak-typed nuclear fusion reactor aimed to the high performance plasma under steady-state condition. The basic design parameters of KSTAR are as follows: *B*_T_ = 3.5 T, *I*_p_ = 2 MA, the major radius *R* = 1.8 m, the minor radius *a* = 0.5 m, the elongation *κ* = 2, and the triangularity δ = 0.8. The NBI, whose direction is clockwise when viewed from above, and the electron cyclotron heating (ECH) are the main plasma heating systems in the KSTAR. In this experiment, plasma is only heated by NBI and central electron temperature is in range of 3~4 keV. Any external perturbations such as supersonic molecular beam injection (SMBI) and resonant magnetic perturbations (RMP) are not applied in this plasma. Various diagnostics such as millimeter wave imaging diagnostics, magnetic diagnostics, and profile measurement diagnostics have been installed in KSTAR. In order to analyze the solitary perturbation, we mainly use the data from the ECEI system, the toroidal Mirnov coil array, and the CES system.

### Electron cyclotron emission imaging system

The ECEI systems provide measurements of the radiation temperature dynamics of SP in poloidal cross-section of the tokamak. There are two independent ECEI systems toroidally separated by 1/16^th^ of the torus (or 22.5 degrees) for 2D and quasi-3D visualization of MHD instabilities in the KSTAR. The 1^st^ ECEI system consists of two detector arrays measuring two different radial view positions at the same time and the 2^nd^ ECEI system has one detector array. Each detector array consists of 24 vertical channels and 8 radial channels with good spatial resolution ~1–2 cm and temporal resolution ~1 *μ*s sufficient for visualization of rapid MHD phenomena such as ELM crash and SP.

### Toroidal mode number estimation technique

The toroidal mode number estimation technique using the 3D ECEI system is applied in this paper. This method can measure the *n* beyond the measurement limitation of the toroidal Mirnov coil array in the KSTAR. This technique involves the measurement of the poloidal wavelength of the mode and of the pitch angle of the mode at the outboard mid plane. 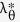
can be estimated by ECEI snapshot and α* can be determined by vertical distance of the same flux tube in the 3D ECEI image or reconstructed by EFIT code.

## Additional Information

**How to cite this article**: Lee, J. E. *et al*. Solitary perturbations in the steep boundary of magnetized toroidal plasma. *Sci. Rep.* **7**, 45075; doi: 10.1038/srep45075 (2017).

**Publisher's note:** Springer Nature remains neutral with regard to jurisdictional claims in published maps and institutional affiliations.

## Supplementary Material

Supplementary Video

Video Legend
