# Supplementary material for: Solitary perturbations in the steep boundary of magnetized toroidal plasma
Source: Sci Rep. 2017 Mar 24;7:45075. doi: 10.1038/srep45075 (PMC5364482; doi:10.1038/srep45075)
Supplement: Video Legend [file srep45075-s2.pdf]

Solitary perturbations in the steep boundary of magnetized toroidal plasma

J. E. Lee[1], G. S. Yun[1*], W. Lee[2], M. H. Kim[1], M. Choi[2], J. Lee[3], M. Kim[3], H. K. Park[2,3], J. G. Bak[2], W. H. Ko[2], and Y. S. Park[4]

[1]*Pohang University of Science and Technology, Pohang 790-784, Korea*

[2]*National Fusion Research Institute, Daejeon 34133, Korea*

[3]*Ulsan National Institute of Science and Technology, Ulsan 689-798, Korea*

[4]*Columbia University, New York, New York 10027, USA*

*Correspondence should be addressed to G.S. Yun (gunsu@postech.ac.kr)

Supplementary video. Dynamics of solitary perturbation (KSTAR shot no. 13250). The top left panel is the time trace of the radiation temperature fluctuations measured by a mid-plane ECEI channel (G1406 with the corresponding $[\mathrm{R}, \mathrm{z}] \approx [216\ \mathrm{cm},\ 3\ \mathrm{cm}]$). The top right panel is a zoomed view around the appearance of SP indicated by the dotted lines on the left panel. The video shows two distinct filamentary structures: a QSM (rotating in the clockwise direction from the top) and a SP (rotating in the counter-clockwise direction from the bottom). It appears that the QSM coalesces into SP or diminishes.
